# Investigation into the biomechanics of lumbar spine micro-dynamic pedicle screw

**DOI:** 10.1186/s12891-018-2132-5

**Published:** 2018-07-18

**Authors:** Chuang Liu, Allieu Kamara, Yunhui Yan

**Affiliations:** 1 School of Mechanical Engineering & Automation, Northeastern University, Shenyang, Liaoning 110819 People’s Republic of China; 2 Department of Pediatric Orthopedics, Shengjing Hospital of China Medical University, Shenyang, 110004 Liaoning Province People’s Republic of China

**Keywords:** Micro-dynamic, Pedicle screw, Biomechanical, Finite element study, Range of motion

## Abstract

**Background:**

Numerous reports have shown that rigid spinal fixation contributes to a series of unwanted complications in lumbar fusion procedure. This innovative micro-dynamic pedicle screw study was designed to investigate the biomechanical performance of lumbar implants using numerical simulation technique and biomechanical experiment.

**Methods:**

Instrumented finite element models of three configurations (dynamic fixation, rigid fixation and hybrid fixation) using a functional L3-L4 lumbar unit were developed, to compare the range of motion of the lumbar spine and stress values on the endplate and implants. An in vitro experiment was simultaneously conducted using 18 intact porcine lumbar spines and segmental motion analyses were performed as well.

**Results:**

Simulation results indicated that the dynamic fixation and the hybrid fixation models respectively increased the range of motion of the lumbar spine by 95 and 60% in flexion and by 83 and 55% in extension, compared with the rigid fixation model. The use of micro-dynamic pedicle screw led to higher stress on endplates and lower stress on pedicle screws. The outcome of the in vitro experiment demonstrated that the micro-dynamic pedicle screw could provide better range of motion at the instrumented segments than a rigid fixation.

**Conclusion:**

The micro-dynamic pedicle screw has the advantage of providing better range of motion than conventional pedicle screw in flexion-extension, without compromising stabilization, and has the potential of bringing the load transfer behavior of fusional segment closer to normal and also lowers the stress values of pedicle screws.

## Background

Treatment with rigid fixation and spinal fusion has been regarded as the gold standard for spine surgery. Traditional transpedicular rigid fixation is widely accepted in clinical practice not only because it is easier and safer, but it also offers better biomechanic stability [1, 2]. From a mechanical perspective, conventional rigid pedicle screws provide over stability leading to stiffness of instrumented segments, stress concentration of implants and stress shielding of the interbody space. Limitations inherent to rigid fixation above may contribute to acceleration of adjacent segment degeneration (ASD) and loosening and rupture of internal fixators [3, 4].

**Fig. 1:**
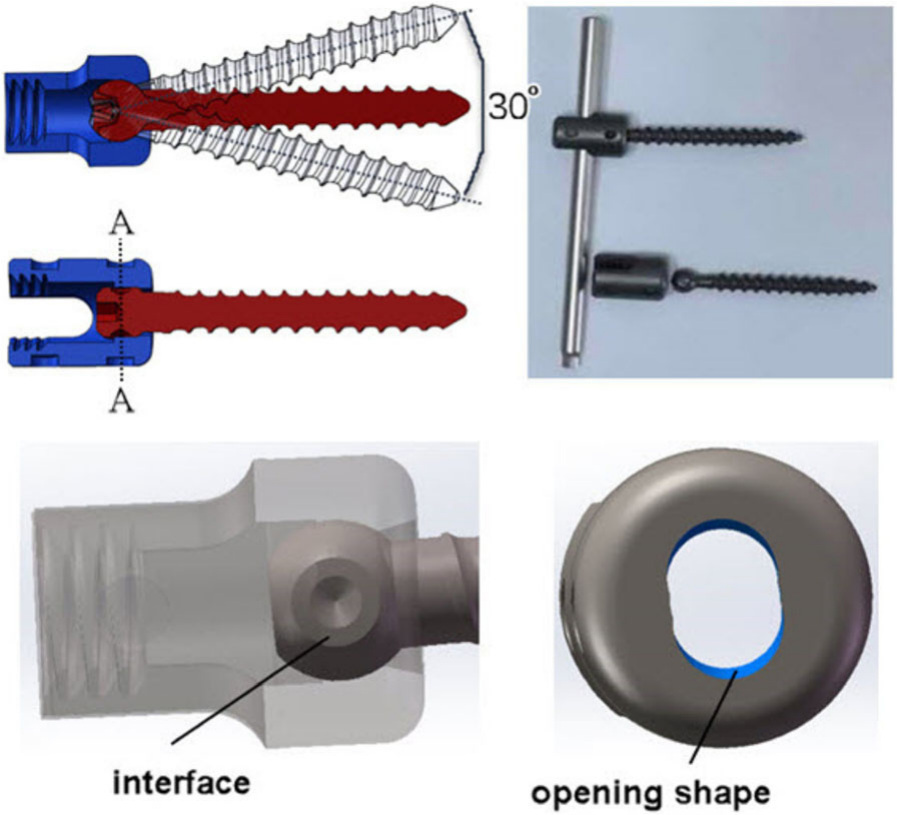
The movable structure of the micro-dynamic pedicle screw and design details

To address the problems caused by rigid fixation, motion preservation techniques have gradually emerged, which may stabilize the bridged segments and preserve the segment motion to a certain extent. The basic mechanics of dynamic stabilization devices is to reduce stiffness of the instrumentation to allow for more physiologic load transmission at the instrumented levels [5, 6]. At present, the mechanism of various dynamic implant designs currently available is mainly based on two components: one is the rod, the other is the pedicle screw. Dynamic systems based on the rod, such as Isobar TTL, PEEK Rod System and Bioflex System, have achieved good clinical results [7–10]. Studies focused on dynamic designs based on the pedicle screw are scant. The most representative one is the Cosmic system, which features a hinge joint between the head and the threaded part. The position of the Cosmic hinge joint could not protect from screws fracture especially because of stress concentration on the root of the screw. Fogel et al. have verified that structure of coupling the screw head to the ball-socket forms a protective feature of the pedicle screw, preventing pedicle screw breakage [11]. In this paper, a micro-dynamic pedicle screw which fully utilizes the advantages of ball and socket joint and Cosmic hinge joint was designed and biomechanically tested. The key design was to deform the ball and the ball socket to generate pinned structure that accommodates sagittal angle variations of the pedicle screws with respect to rods, in order to allow articulation and flexibility after locking of the screw-rod joint. Figure 1 illustrates the movable structure of the micro-dynamic pedicle screw and a physical photograph. The purpose of this study was to characterize the biomechanical properties of the micro-dynamic pedicle screw (MDPS) for the lumbar spine to compare its kinematic and biomechanical performance against that of traditional rigid pedicle screws (PS). Table 1 was the PICO framework of this study.

**Table 1:** PICO framework of the study

Patient/Problem	Intervention	Comparison	Outcome
Patients undergoing lumbar rigid fixation and fusion/ Acceleration of adjacent segment degeneration (ASD) and loosening and rupture of internal fixators	Dynamic stabilization using micro-dynamic pedicle screws	Traditional rigid pedicle screws	ROM of the surgical level, stress on the endplate, stresses on the implants

## Methods

The micro-dynamic pedicle screw (MDPS) is a novel design where the uniaxial head coupling to the screw forms a screw-hinge joint. The screws are used to anchor the anterior vertebral bodies with the uniaxial head and then linked by titanium rods. This do not only have the ability of reducing pathologic motion or hypermobility of the instrumented spine, but also can preserve moderate motion at the instrumented levels. In this paper, both in vitro animal experiments and finite element (FE) analysis techniques were employed to gain insight into the lumbar biomechanics of MDPS, in comparison to clinically proven rigid systems. This research involving human data and animal specimens has been approved by Medical Ethics Committee, Shengjing Hospital of China Medical University, and the reference number is 2017PS059K.

### Design of the MDPS

Since subtle ROM for lumbar spine fusion in flexion-extension may have unexpected positive effects, the overall design philosophy of MDPS is to achieve an approximate release of angle constraint in the sagittal plane. The first step was to create a functional interface between screw seat and screw ball that provides minimal motion of the pedicle screws in the sagittal plane, with respect to rods after locking of the screw. The opening shape of the screw seat can give a good angular-control ability of the pedicle screw. A limited angle was initially set at 30°, based on the experiences of predecessors [12]. However, the optimum angle range still needs further study. The movable structure and design details of the micro-dynamic pedicle screw are shown in Fig. 1.

### Finite element model of lumbar vertebra

This study was supported by Shengjing Hospital of China Medical University. A detailed, anatomically accurate three-dimensional non-linear finite element model(FEM) of L3-L4 spine was first developed. The vertebral geometry data for the intact lumbar spine were obtained from a 0.5 mm thick, transverse slices CT scans of a 25-year-old healthy male volunteer. Briefly, the model contained the functional spinal unit L3-L4 with tension-only, linear ligaments, incompressible nucleus pulposus and surface-to-surface non-linear frictional contact in the facets. The vertebral bodies were composed of a cancellous bone core, a 0.5-mm thick cortical shell surrounding and posterior bone regions. The intervertebral disc was modeled as a composite structure including annulus fibrosis and nucleus pulposus. All the six major ligaments, interspinous (ISL), supraspinous (SSL), intertransverse (ITL), posterior longitudinal (PLL), anterior longitudinal (ALL) and ligamentum flavum (LF) were simulated in the model. Besides, the upper and lower endplates with 1-mm thickness were constructed lying at the interlamination between vertebral body and intervertebral disc [13, 14]. All the components of the vertebrae were considered as isotropic homogeneous elastic materials. Material properties were selected from previous FE studies as listed in Table 2 [14]. All the FEM pre-processing works were completed in Hypermesh 12.0.

**Table 2:** Material Property Designations of the Finite Element Model

Component	E (MPa)	ʋ	Cross-Sectional Area(mm)
Cortical	12,000	0.3	–
Cancellous	100	0.2	–
Posterior	3500	0.25	–
Fibrosus	450	0.3	–
Pulposus	1	0.499	–
Endplate	23.8	0.4	–
Ligaments	–	–	–
ALL	7.8(ε < 0.12)	0.45	67.3
	20(ε > 0.12)		
PLL	10(ε < 0.11)	0.45	20
	20(ε > 0.11)		
FL	15(ε < 0.062)	0.45	40
	19.5(ε > 0.062)		
SSL	8(ε < 0.20)	0.45	30
	15(ε > 0.20)		
ISL	10(ε < 0.14)	0.45	40
	11.6(ε > 0.14)		
ITL	10(ε < 0.18)	0.45	18
	58.7(ε > 0.18)		
Implants	–	–	–
Titanium	110,000	0.33	–

**Fig. 2:**
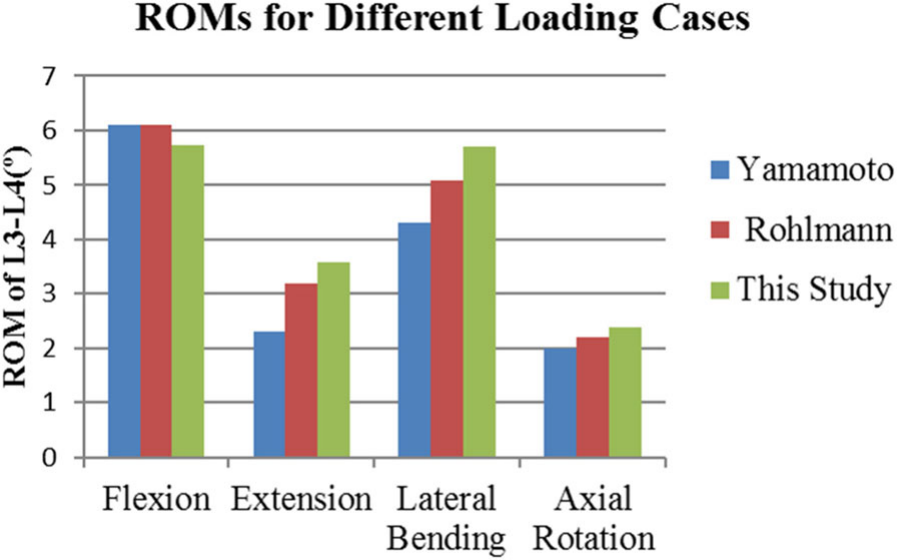
Comparison of predicted results by the current FE model against investigations by Yamamoto and Rohlmann. The applied moment was 10 Nm

Before utilizing this model to evaluate the biomechanical performance of MDPS, its validation had to be operated via comparing the predicted data obtained by the current model with the results of in vitro tests performed by Yamamoto as well as the results of FE tests performed by Rohlmann [15, 16]. The FE model was exported to commercial FE software (ABAQUS6.14; Hibbitt, Karlsson& Sorenson, Inc., Providence, RI, USA) to simulate physiologic motion of the segments in extension (E), flexion (F), lateral bending (LB) and axial rotation (AR), with the boundary and loading conditions being kept consistent with the literatures. Figure 2 shows the range of motion (ROM) predicted by the model, matched well with the data from the literatures.

**Fig. 3:**
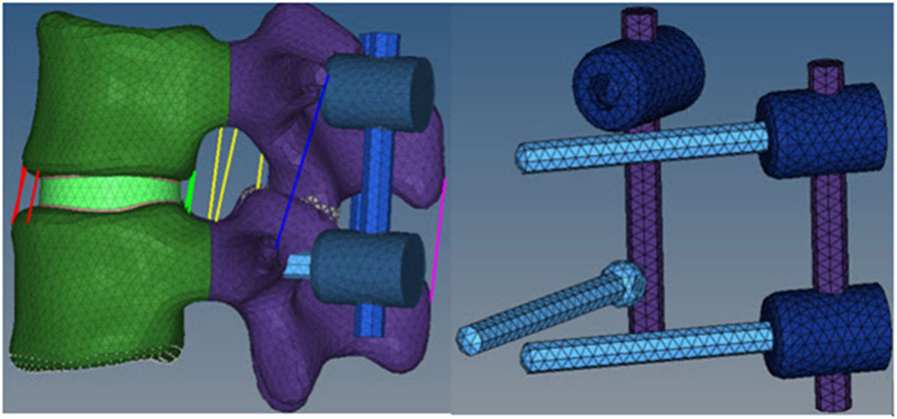
The FEM of instrumented L3-L4 and MDPS

**Fig. 4:**
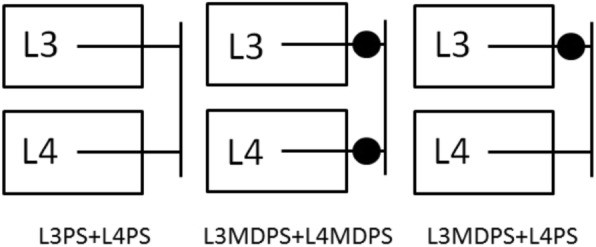
Sketches of the three configurations instrumented models

The FEM of intact L3-L4 segment was employed to develop the instrumented models, as shown in Fig. 3. The associated parameters of geometric size and material strength were modified to simulate as moderate degeneration [17]. Three configurations (L3PS + L4PS, L3MDPS + L4MDPS, L3MDPS + L4PS) illustrated in Fig. 4 were developed to help understand the biomechanics of MDPS. The geometric size of the rods and screws of all fixators were the same for equivalent comparison. The instrumented models were analyzed under the same boundary condition of fixing in all degrees of freedom at the bottom of L4 and same loading condition of 10 Nm bending moment applied in flexion, extension, lateral bending, and axial rotation on the top of L3. The ROMs which were the primary parameter reported in the biomechanical evaluation of models were compared among the three configurations. The stress on the endplates were also recorded as dynamic stabilization devices have the advantages of reducing stress shielding. In addition, stress on the instrument plays an important role in our simulation because of its direct relation to the endurance limit of the implants, therefore, stress on the rods and pedicle screws were also investigated for this reason.

### In vitro animal experiments

In vitro studies are necessary to support the biomechanical findings obtained from finite element analysis (FEA) technique. The animal experiments were set up to investigate the feasibility and safety of MDPS as well as investigate the ROMs of the three configurations (L3MDPS + L4MDPS, L3PS + L4PS, L3MDPS + L4PS) instrumented models using a pure-moment flexibility testing protocol. The experiments were performed at the minimally invasive medical device engineering research center of Ministry of Education at Shanghai University of Science and Technology. The specimens were purchased from the local livestock farmers. During this matter,animal welfare and experimental procedures were carried out in accordance with the Guide for the Care and Use of Laboratory Animals (Ministry of Science and Technology of China, 2017), and were approved by the animal ethics committee of Shengjing Hospital of China Medical University.

**Fig. 5:**
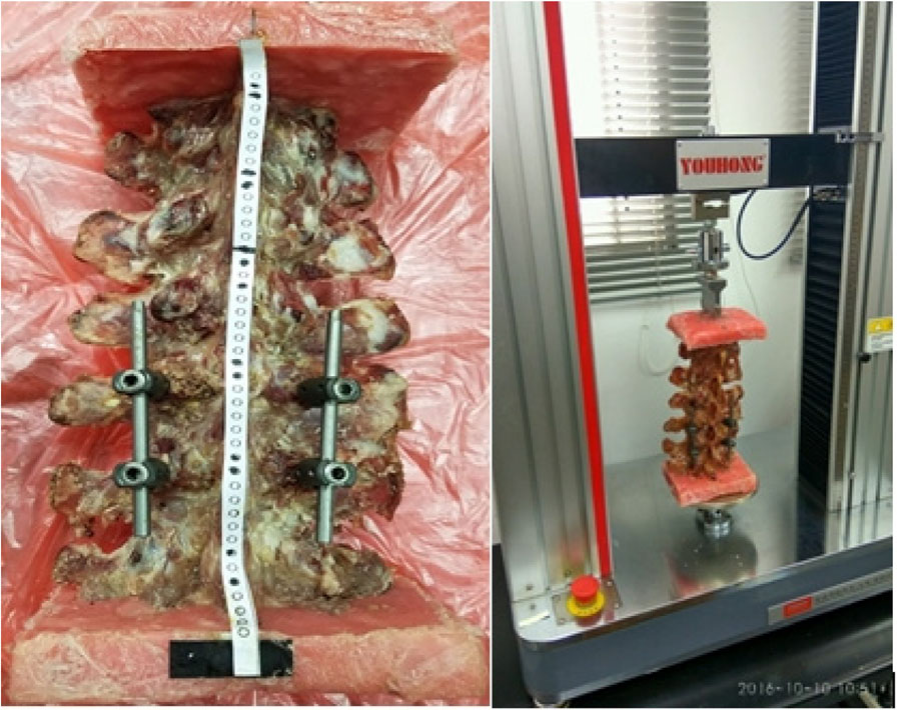
Customized optical markers were firmly attached to the anterior surface of the vertebral body. Porcine lumbar spine specimens were mounted in a hydraulically actuated spinal loading machine capable of applying pure moments in flexion, extension and lateral bending

Eighteen fresh-frozen porcine lumbar spine specimens with L1 to L5 segment were used in this investigation. Each specimen was radiographically imaged, to exclude specimens demonstrating any anomalies or fractures. The specimens were thawed at room temperature, and the tissue surrounding the spine, except the ligaments and facet joints were carefully removed. Each spine was embedded in a self-solidifying dental base acrylic resin liquid and dental base acrylic resin powder cranially at L1 and caudally at L5 to get smooth end faces. Finally, optical markers were firmly attached to the anterior surface of each vertebral body to check for any posttest deformity, and then tested in the four loading directions. Figure 5 shows the processed specimens and the loading process.

First, we randomly selected six uninstrumented spines and tested in the four loading directions to determine the baseline ROM. Subsequently, eighteen specimens were equally divided into three groups to perform instrumentation according to the three configurations (L3MDPS + L4MDPS, L3PS + L4PS, L3MDPS + L4PS). Spines were regularly sprayed with normal saline to prevent moisture loss until all tests was completed. Multiple comparisons of the obtained ROMs of the implanted segment for each of the three configurations were made using paired t-tests for each loading scheme (F, E, LB and RB). Significance levels was set at *P* < 0.05. Statistical analyses was performed using SPSS Statistics, version 23.0 (IBM Corp, Armonk, NY).

## Results

### FE simulation analysis: ROM data, stresses on endplates and implants

**Fig. 6:**
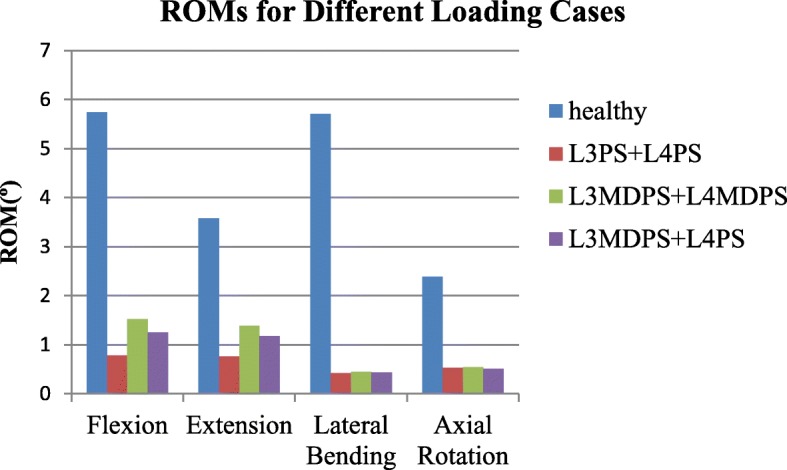
ROM of L3-L4 calculated for the lumbar spine finite element model in the healthy and three instrumented configurations

The dynamic fixator MDPS is designed to increase the ROM appropriately without compromising stability. ROM of L3-L4 motion segments for the three configurations are summarized in Fig. 6. In all loading conditions, each configuration instrumented model demonstrated a significant reduction in segmental ROM compared to the intact model. In flexion and extension, the L3MDPS + L4MDPS model ROM increased by 95 and 83% respectively, while that for the L3MDPS + L4PS model increased by 60 and 55% respectively, when compared with the L3PS + L4PS model. The ROMs for the three configurations models in lateral bending and axial rotation were similar, which was consistent with our expected result.

The stress shielding effect during transition was evaluated in terms of stress on the endplate. Compared with L3PS + L4PS model, the endplate stress in the L3MDPS + L4MDPS model increased by 6.5, 4.5, 4.8, and 8.0%in flexion, extension, lateral bending, and axial rotation respectively, while that of the L3MDPS + L4PS model increased by 2.3, 0.4, 3.7, and 4.0%, respectively.

Stresses on the implants were also quantified. Compared to the L3PS + L4PS model, the L3MDPS + L4MDPS model had decreased maximum Von Mises stress (MVMS) on screws in flexion, extension and lateral bending, which decreased by 15.9, 16.2 and 9.2% respectively, but increased on screws in axial rotation by 22.6%. Meanwhile, the L3MDPS + L4MDPS model had increased MVMS on rods in all loading conditions. The L3MDPS + L4PS model demonstrated intermediate values of MVMS on screws and rods, between the other two models.

### In vitro experiment: ROM data

**Fig. 7:**
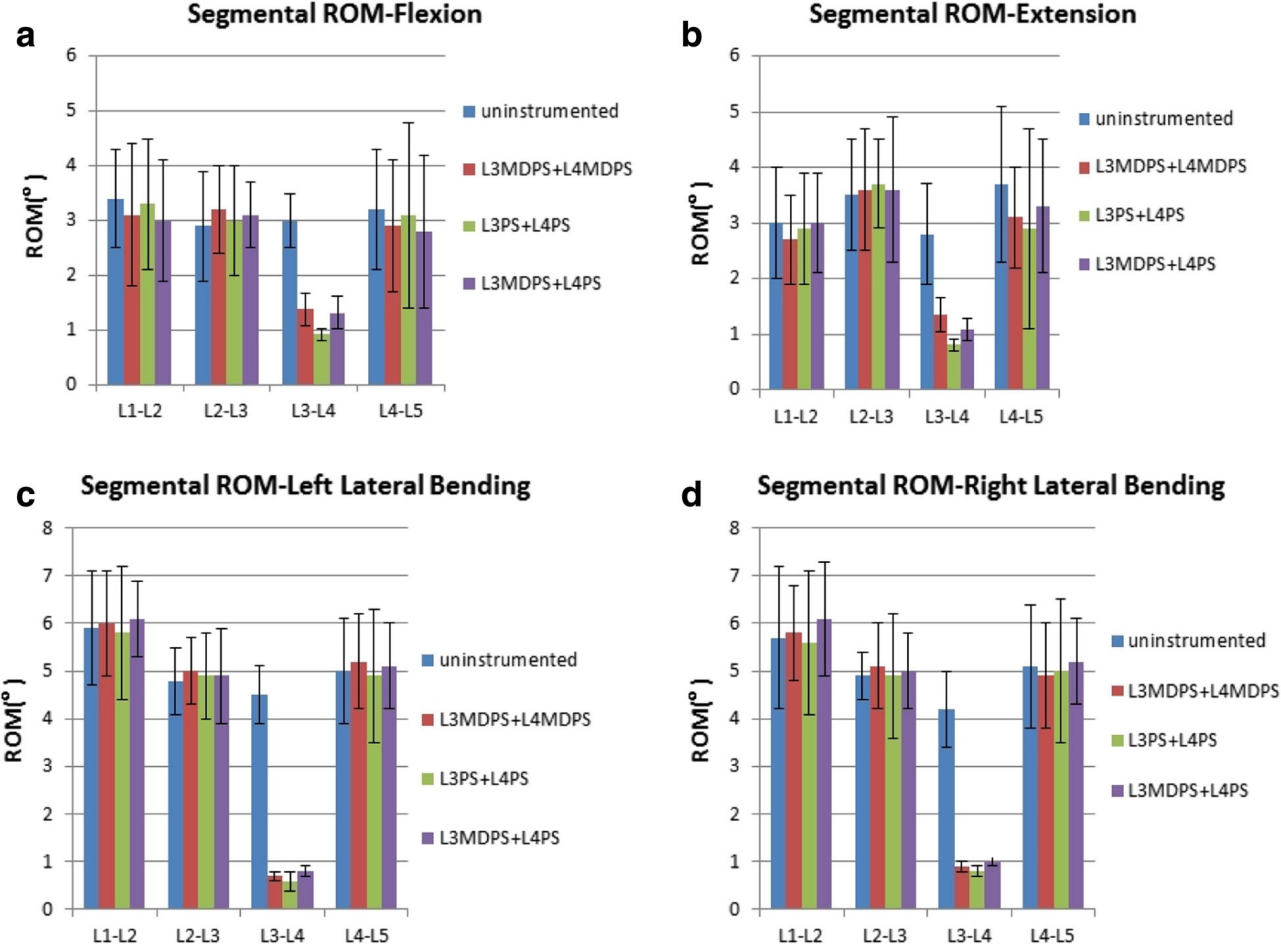
Comparison of each segmental ROM data showing mean ROM ± SD from L1-L5 for each group specimens. **a** Flexion. **b** Extension. **c** Left Bending. **d** Right Bending

The mean ROM of each level for all grouped specimens in F, E, LB and RB are shown graphically in Fig. 7. From the overall perspective, ROM values of unimplanted levels for all specimens were rather similar, while instrumentation of L3-L4 led to a statistically significant decreased ROM in that segment. The comparison was concentrated on the ROM of L3-L4. A statistically significant increase in ROM was observed between the L3PS + L4PS and L3MDPS + L4MDPS groups and between the L3PS + L4PS and L3MDPS + L4PS groups in both flexion and extension (Tables 3).

**Table 3:** Group statistics and independent samples test between specimens of the L3PS + L4PS and the L3MDPS + L4MDPS, and between specimens of the L3PS + L4PS and the L3MDPS + L4PS

Group Statistics and Independent Samples Test						
Load	Models	N	Mean	Std. Deviation	Std. Error Mean	Sig. (2-tailed)
Flexion	L3PS + L4PS	6	.9235	.11014	.04496	0.029
	L3MDPS + L4MDPS	6	1.3885	.38074	.15544	
Extension	L3PS + L4PS	6	.8051	.08874	.03623	0.01
	L3MDPS + L4MDPS	6	1.3654	.34830	.14219	
Flexion	L3PS + L4PS	6	.9235	.11014	.04496	0.017
	L3MDPS + L4PS	6	1.3270	.32559	.13292	
Extension	L3PS + L4PS	6	.8051	.08874	.03623	0.008
	L3MDPS + L4PS	6	1.0885	.19233	.07852	

## Discussion

### Motion-preservation design

A limited number of studies have investigated dynamic devices based on dynamic pedicle screw. The MDPS was designed to preserve index level mobility and thereby avoiding the cascade of adjacent segment degeneration (ASD). Cakir et al. reported in their study that increase segmental ROM in flexion/extension was positively correlated with better clinical outcome [18]. Therefore, the MDPS is superior to others because of the loosening degrees of freedom (DOF) allowed in flexion-extension. The hinge joint allows ±15° of movement about the A-A axis (Fig. 1) [19]. Compared to Cosmic screw which features a hinge joint between the head and the threaded part, the improvement of MDPS is that the hinge joint is generated inside the ball and socket. This coupling structure may protect the pedicle screw from breaking when it suffers an accident [11].

The FE results of ROM control showed that all three patterns of fixation could provide adequate stabilization at the instrumented segments. The difference was that the introduction of MDPS increased the ROM in flexion and extension. In comparison with theL3PS + L4PS model, the L3MDPS + L4MDPS model increased the ROM by 95 and 83% in flexion and extension respectively. This finding suggests that, MDPS may offer better motion preservation at implanted segment. Although the increased ROM value was relatively small in both flexion and extension (0.74° and 0.63° respectively), which is due to the moderate design, this small change can induce significant alteration of the mechanical environment of the segmental fusion [20, 21].

### Stresses on endplates and implants

**Fig. 8:**
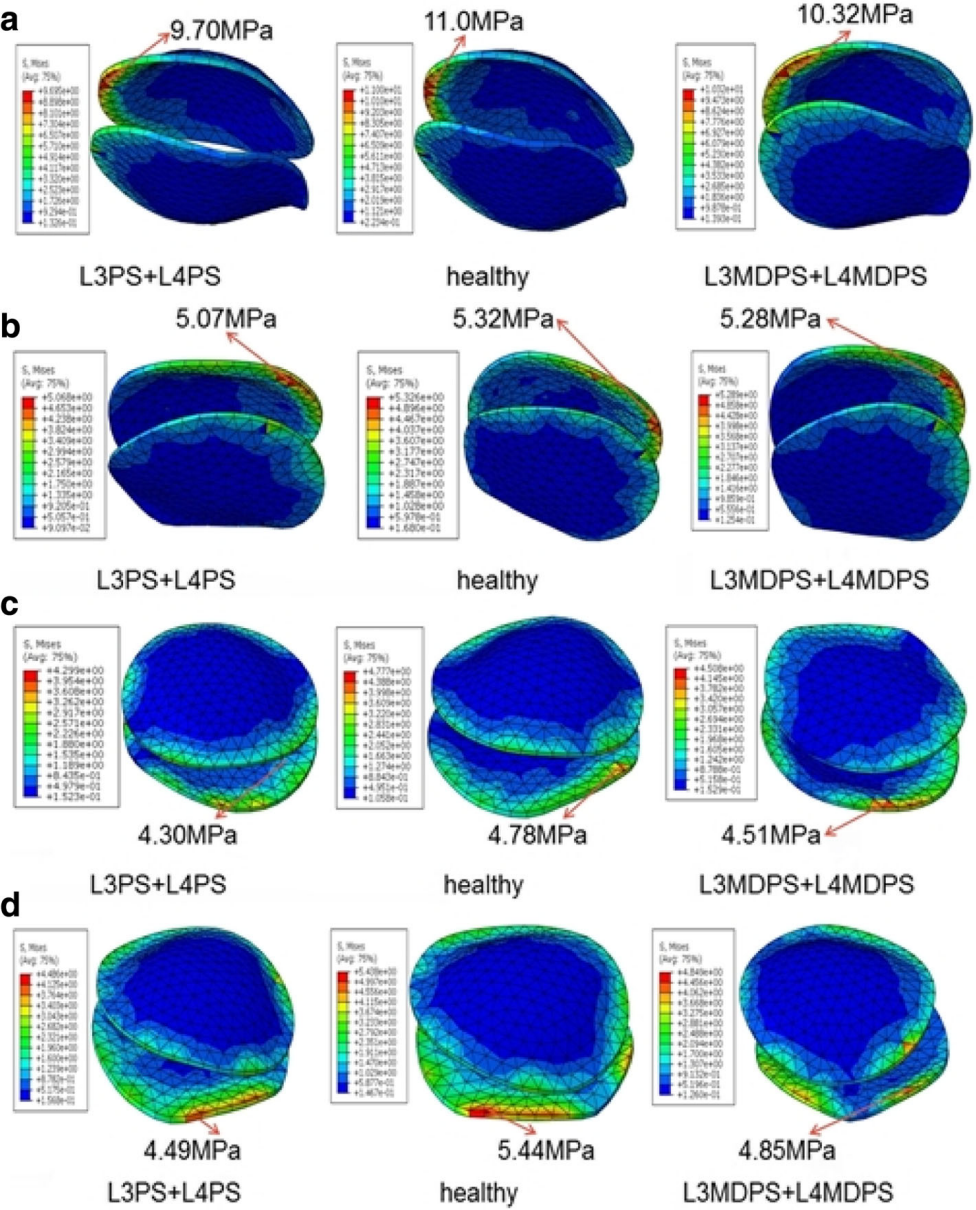
Maximum stress of endplate for the L3PS + L4PS, healthy and L3MDPS + L4MDPS models when tested in (**a**), flexion; **b** extension; **c** lateral bending; and (**d**) axial rotation after applying a moment of 10 Nm

For years, rigid fixation has been proven to lead to adverse stress-shielding effects of the interbody space, on account of excessive stabilization [22]. The MDPS allowed more mobility and created an improved environment for load transfer among vertebral bodies, endplates and lumbar discs than the conventional screws. Von Mises equivalent stress of endplates for the healthy uninstrumented spine, L3PS + L4PS and L3MDPS + L4MDPS models are shown graphically in Fig. 8. For the healthy uninstrumented model, the peak stress of endplates was 11 MPa in flexion, and 5.33 MPa in extension. This simulation result was very similar to those of previously published study by Goto (9.43 MPa in F, 4.70 MPa in E) [23]. Compared to the intact model, the decreased endplate stress of the L3PS + L4PS and L3MDPS + L4MDPS model were as follows: 11.9 and 6.2% in flexion, 5 and 0.75% in extension, 10 and 5.6% in lateral bending and 15.6 and 10.8% in axial rotation, respectively. The peak stress of endplates in the MDPS models were very close to that of the intact model. It could be inferred that the small increase of ROM induced by MDPS made the load transfer behavior of fusional segment more close to that of normal lumbar spine. Shirado et al. found in their specimens of severely degenerated disc, very low stresses at the endplate [24]. The L3PS + L4PS model in our study also showed low stresses at the endplate. Another fact is that, rigid fixator may cause abnormal changes in the load transfer that may lead to degeneration of the intervertebral disc and bony structures [25], and consequently leading to low endplate stress. Consequently, from a logical point of view, the MDPS has the advantage of promoting load transfer between the bridged segments and thereby reducing stress shielding effect over rigid pedicle screw fixation.

**Fig. 9:**
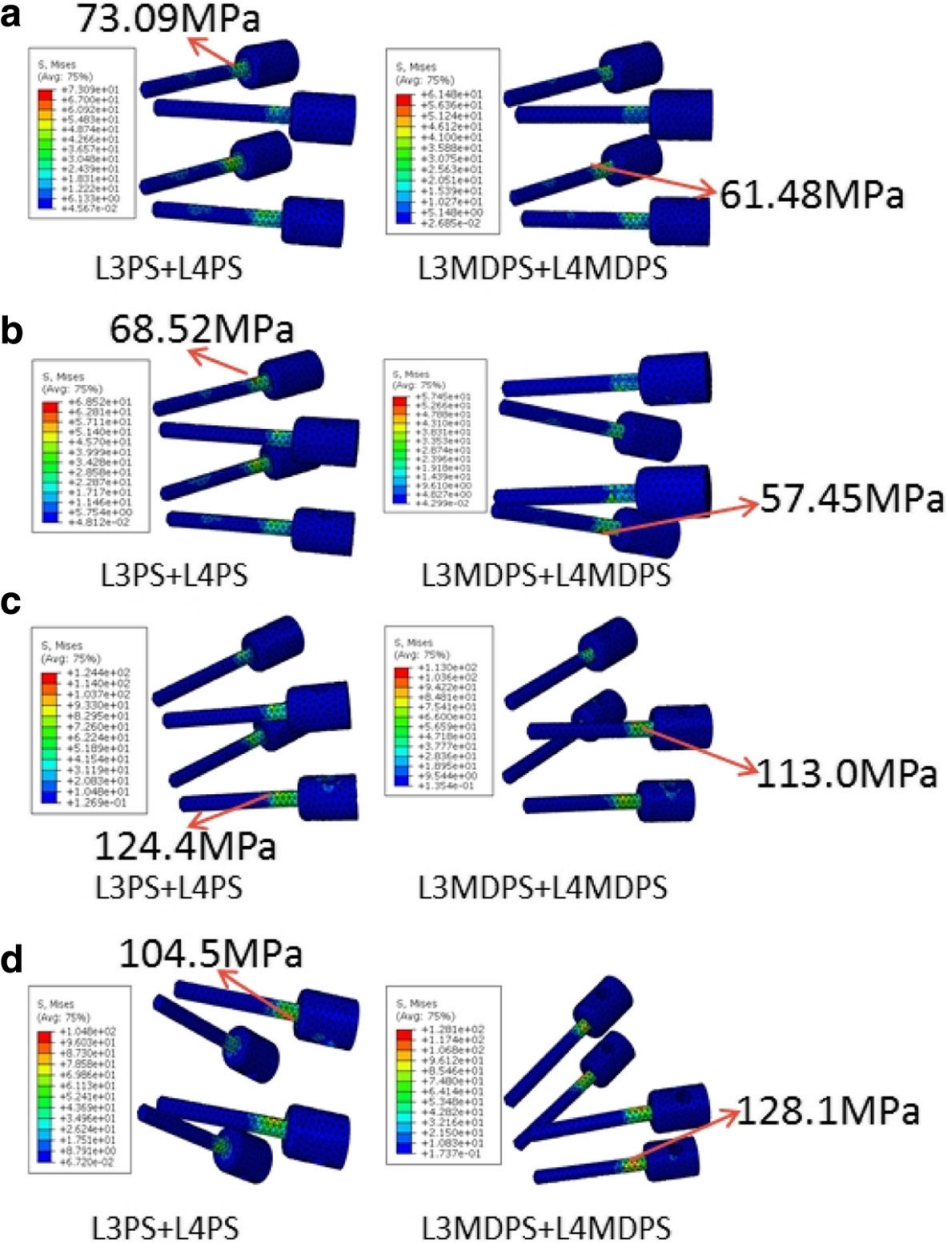
Maximum stress of screws for the L3PS + L4PS and L3MDPS + L4MDPS models when tested in (**a**) flexion; **b** extension; **c** lateral bending; and (**d**) axial rotation after applying a moment of 10 Nm

**Fig. 10:**
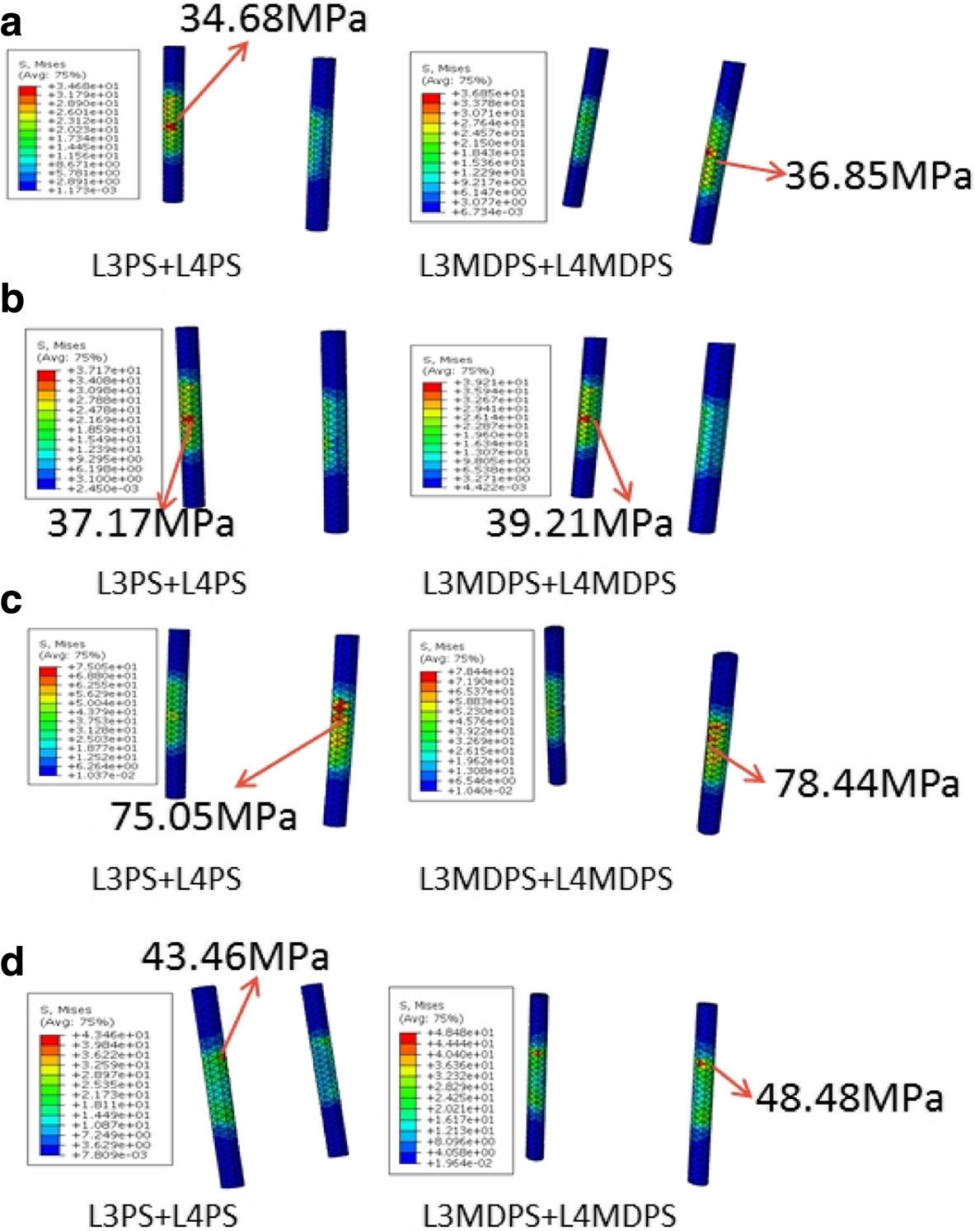
Maximum stress of rods for the L3PS + L4PS and L3MDPS + L4MDPS models when tested in (**a**) flexion; **b** extension; **c** lateral bending; and (**d**) axial rotation after applying a moment of 10 Nm

Pedicle screw systems have to provide segmental stabilization and load transfer throughout its life. During this period, they would be subjected to constant cyclic dynamic loading after surgical operation. Implant failure is an important consideration to make when designing an implant. The stress level on the implant has a significant impact on its endurance limit as well. Von Mises equivalent stresses of the pedicle screws and rods for the L3PS + L4PS versus L3MDPS + L4MDPS models are shown graphically in Fig. 9 and Fig. 10, respectively. For flexion and extension, the L3MDPS + L4MDPS device lowered the MVMS of screws by about 16%, as compared to those of the L3PS + L4PS device (Fig. 9a and Fig. 9b). This could be due to the fact that, MDPS decreased the spine system’s bending rigidity, since it was given slight motion relative to the rod in sagittal plane. This motivation improved the stress concentration on the pedicle screw in conventional rigid fixation. For lateral bending, the MVMS of screws for the L3MDPS + L4MDPS device was lower by 9.2% when compared to the L3PS + L4PS device (Fig. 9c). However, when comparing the two systems in axial rotation, L3MDPS + L4MDPS increased the MVMS of screws by 22.2% (Fig. 9d). This was probably because the ball screw was absolutely restricted by the socket in the transverse plane, resulting in more significant interference between the MDPS and vertebrae in axial rotation. Lower stress values of pedicle screws for dynamic stabilization devices was also observed by Ahn et al. [26]. They drew the inference that, higher axial load was transmitted across the anterior structure with the dynamic devices. We believe that, the dynamic flexibility of the device may perform a little better if the load distribution between screws and rods is homogenized.

### ROM data of porcine spine

Because of the limitation by test conditions, we only investigated the ROMs of specimens only in this study. There was some difference between our test results and previously reported data, may be owing to the different experimental conditions and experimental methods used. A study about quantitative biomechanical properties of the whole porcine was conducted by Wilke et al. [27]. The ROMs of the motion segments of the intact porcine lumbar in our current study were a slightly lower, when compared with Wilke’s. However, both results followed the same trend, and data showed the lowest value at the L3-L4 segment. In an in vitro experiment of calf spine lumbar segments, Scifert et al. compared the biomechanical stabilization of a rigid versus a dynamic posterior fixation device [19]. Their results indicated a 5.6% increase in motion in flexion and a 6.2% increase in extension across the destabilized level for dynamic pedicle screw device, compared to rigid pedicle screw device normalized to the static model. The corresponding results in our experiment were: a 20% increase in flexion and a 21.4% increase in extension. The differences may be attributable to the anatomy, size or material characteristics of vertebral body between pig and calf. However, this outcome demonstrated that MDPS can provide larger ROM at the instrumented segments than a rigid one.

### Clinical considerations

Posterior dynamic stabilization (PDS) is now a popular form of surgical intervention, which may relieve symptoms of spinal stenosis as well as discogenic pain by reducing pathologic motion and improving the distribution of loads while preserving the integrity of the native disk and facet joints. PDS devices are FDA-approved for use as adjuncts to spinal fusion. The MDPS does not only inherit the merit of design of ball-socket, but also combines the concept of hinge joint of cosmic system. However, the only downside to MDPS is that kinematic in the uniaxial sagittal plane may lead to malalignment between the rod and the fixed-angle screw head. To overcome the problem of uniaxial malalignment between the rod and fixed angle screw head of MDPS, hybrid use with a conventional pedicle screw is recommended. The biomechanical effects of the L3MDPS + L4PS model was intermediate in the comparison, but this pattern provided more freedom on the instrumentation to facilitate easier rod seating into the screw head saddle. Therefore, the hybrid fixation may be a promising alternative option to treat spinal stenosis or chronic low-back pain conservatively. It is of clinical significance that the choice of MDPS and rigid system might depend on the desired stability of the surgical segment. A surgical segment with more severe degeneration can be protected by using the MDPS due to its higher motion and load transfer abilities. In other situations, the rigid system is more appropriate in response to higher joint instability.

### Study limitations

We have to note some potential limitations of the current study. Firstly, the design of ±15° rotational degrees of freedom for MDPS, which plays a significant role in controlling the segmental stability of the spine, stemmed from a former pedicle hinged screw–rod system (Osteotech, Inc., Eatontown, NJ, USA). An in-depth clinical investigation into the angles is lacking in the literature. Therefore, a large accumulation of clinical data to make the support of its role is required. Secondly, the FE study was limited by simplifications in several ways: the use of linear elastic and homogeneous materials for the entire vertebral body and ligaments, tie contact between screw and surrounding bone, absence of the tension of lower-back muscles, and so on. All the above assumption would have fundamental impact on the simulation result. However, the goal of this study was to simply compare the biomechanical stability of the devices, therefore the final results obtained may not have been influenced much by the above limitation. Finally, porcine lumbar spines were employed in the in-vitro biomechanical experiments due to lack of human cadaver specimens. The ROMs data could be limited by the anatomic differences between human and pig. The porcine lumbar spine had little ROM in axial rotation, that may have a notable impact on the biomechanical evaluation of the instrumentations.

## Conclusion

Biomechanical evaluation of a MDPS for spinal fusion has been demonstrated through finite element simulation and in vitro animal experiments. Our results indicate that MDPS has the advantage of providing larger ROM in flexion and extension without compromising stabilization of the bridged segments, as compared to conventional pedicle screw. The dynamic effect of MDPS has the potential to make the load transfer behavior of fusional segments closer to that of normal, and lowers the stress values of pedicle screws.

## Abbreviations

ALL: Anterior longitudinal ligament
AR: Axial rotation
CT: Computed tomography
E: Extension
F: Flexion
FEM: Finite element model
FL: Flavum ligament
ISL: Interspinous ligament
ITL: Intertransverse ligament
LB: Lateral bending
MDPS: Micro-dynamic pedicle screw
PDS: Posterior dynamic stabilization
PLL: Posterior longitudinal ligament
PS: Pedicle screw
ROM: Range of motion
SSL: Supraspinous ligament
